# Solitary Gluteal Subcutaneous Metastasis after Curative Resection of Cecal Cancer: A Rare Presentation Preceding Systemic Metastasis

**DOI:** 10.70352/scrj.cr.26-0096

**Published:** 2026-05-28

**Authors:** Shusaku Hiyama, Ken Imaizumi, Nobuki Ichikawa, Tadashi Yoshida, Yosuke Ohno, Kengo Shibata, Shunji Sano, Honori Okubo, Zenichi Tanei, Shinya Tanaka, Akinobu Taketomi

**Affiliations:** 1 Department of Gastroenterological Surgery I, Graduate School of Medicine, Hokkaido University, Sapporo, Hokkaido, Japan; 2 Department of Surgical Pathology, Hokkaido University Hospital, Sapporo, Hokkaido, Japan

**Keywords:** subcutaneous metastasis, gluteal or perineal region, colon cancer

## Abstract

**INTRODUCTION:**

Cutaneous and subcutaneous colon cancer metastases to the gluteal or perineal regions are extremely rare. Unlike the local recurrence or tumor implantation observed in rectal cancer, gluteal or perineal colon cancer metastasis likely represents a distinct pathophysiological mechanism. We report a case of a solitary subcutaneous gluteal metastasis that developed after curative resection of cecal cancer.

**CASE PRESENTATION:**

A 58-year-old woman underwent laparoscopic ileocecal resection with D3 lymph node dissection for a stage IIIC cecal adenocarcinoma. Postoperative adjuvant chemotherapy with capecitabine and oxaliplatin (CAPOX) was administered for 4 cycles but discontinued owing to hepatic dysfunction. Nine months postoperatively, the tumor marker levels increased; imaging revealed a subcutaneous mass in the left gluteal region. The gluteal mass was completely resected with clear margins. The histopathological findings were consistent with metastasis from the previously resected cecal carcinoma. Adjuvant CAPOX was resumed for 4 additional cycles. Nine months after reoperation, the patient developed a solitary brain metastasis, which was surgically removed, followed by radiotherapy. Subsequently, systemic chemotherapy was initiated, and the patient remains alive without evidence of recurrence 5 months after resection of the brain metastasis.

**CONCLUSIONS:**

Gluteal or perineal subcutaneous metastases from colon cancer are rare and may represent an early manifestation of systemic disease rather than an isolated metastatic event. Even after curative resection, careful postoperative surveillance is warranted.

## Abbreviations

CAPOX
: capecitabine and oxaliplatin
CEA
: carcinoembryonic antigen
FDG
: fluorodeoxyglucose

## INTRODUCTION

Subcutaneous metastasis is an uncommon manifestation of distant colorectal cancer spread,^1)^ and metastasis to the gluteal or perineal regions is extremely rare. Most reported cases of perineal subcutaneous metastasis result from local recurrence and tumor implantation of rectal cancer,^2,3)^ whereas colon cancer metastasis to the gluteal or perineal regions likely represents a distinct pathological mechanism. A previous case report has described a clinical course similar to that of the present patient,^4)^ providing valuable insights into potential metastatic pathways. Here, we report a case of solitary subcutaneous gluteal metastasis that developed after curative resection of cecal cancer.

## CASE PRESENTATION

A 58-year-old Japanese female presented to her primary physician with a positive fecal occult blood test. Further examination led to the diagnosis of cecal cancer, and she was referred to our department for surgical treatment. A laparoscopic ileocecal resection with D3 lymph node dissection was performed. Histopathological findings revealed an ulcerative adenocarcinoma that was predominantly well-differentiated, with focal moderately and poorly differentiated components, invading the serosa (pT4a). Moderate lymphatic invasion and mild venous invasion were observed, whereas no perineural invasion was identified. All resection margins were negative. Metastases were identified in 7 of 14 regional lymph nodes (pN2b), corresponding to pathological stage IIIC. Molecular analysis revealed a *RAS* mutation, wild-type *BRAF*, and low microsatellite instability. Postoperative adjuvant chemotherapy with CAPOX was planned for 6 months but was discontinued after 4 cycles owing to hepatic dysfunction. Nine months after surgery, her CEA was elevated at 7.3 ng/mL, and contrast-enhanced CT revealed a well-defined, irregular 25-mm mass with enhancement in the left gluteal region. ^18^F-FDG PET/CT demonstrated intense FDG uptake at this site (**Fig. 1**), with no other lesions suggestive of metastasis. Therefore, diagnostic and therapeutic excision of the gluteal mass was performed. The tumor margins were confirmed using intraoperative ultrasonography under general anesthesia in the jackknife position. The tumor was resected en bloc with the surrounding adipose tissue, while preserving the external anal sphincter. The operation lasted 46 min and blood loss was negligible. The patient’s postoperative course was uneventful, and she was discharged without complications. Gross examination of the resected specimen revealed a white, solid mass measuring 28 × 28 × 25 mm (**Fig. 2**). Histopathological examination revealed findings similar to those of the previously resected cecal carcinoma (**Fig. 3**), confirming the diagnosis of subcutaneous cecal cancer metastasis. Postoperatively, her serum CEA level decreased to 3.2 ng/mL 1 month after the gluteal mass excision. Because recurrence occurred after discontinuation of CAPOX due to hepatic dysfunction, CAPOX was considered to have potentially contributed to disease control. Accordingly, the remaining 4 cycles of CAPOX, which had not been completed during the initial treatment, were administered after resection of the subcutaneous metastasis with a reduced oxaliplatin dose. Nine months after reoperation, the patient developed right upper limb numbness and dysarthria. Further evaluation revealed brain metastasis from the cecal cancer (**Fig. 4**). The brain lesion was surgically removed, followed by radiotherapy. Subsequently, systemic chemotherapy with folinic acid, fluorouracil, and irinotecan combined with bevacizumab was initiated, and the patient remains alive without evidence of recurrence 5 months since resection of the brain metastases.

**Fig. 1:**
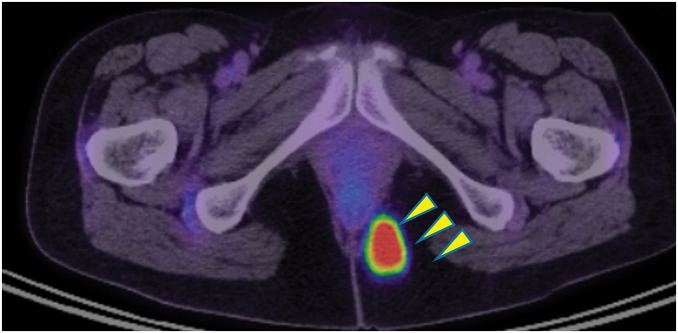
^18^F-FDG PET/CT demonstrated intense FDG uptake in the subcutaneous tissue of the left gluteal region, with a SUV-max of 18.289 (yellow arrowheads). FDG, fluorodeoxyglucose; SUV-max, maximum standardized uptake value

**Fig. 2:**
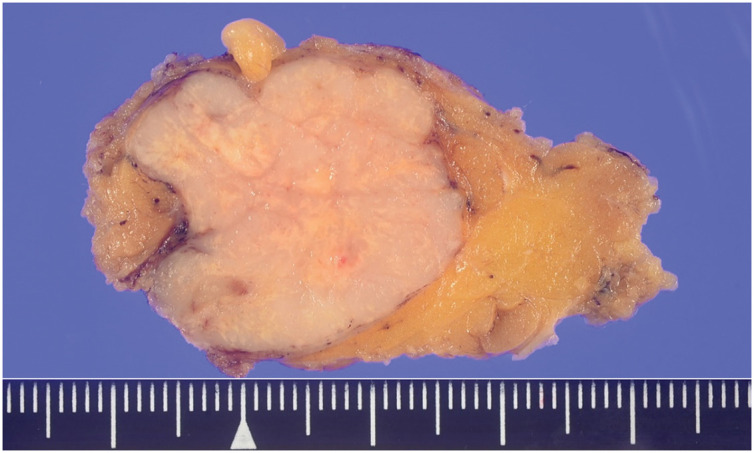
Gross examination of the resected specimen revealed a white, solid mass measuring 28 × 28 × 25 mm.

**Fig. 3:**
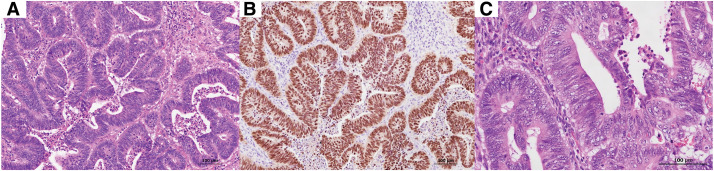
Histopathological findings. (**A**) HE staining of subcutaneous tumor (×40). (**B**) Immunohistochemical staining of the subcutaneous tumor showed CDX-2 positivity (×40). (**C**) HE staining of the primary cecal cancer, showing a similar histological pattern (×100). CDX-2, caudal-type homeobox 2; HE, hematoxylin and eosin

**Fig. 4:**
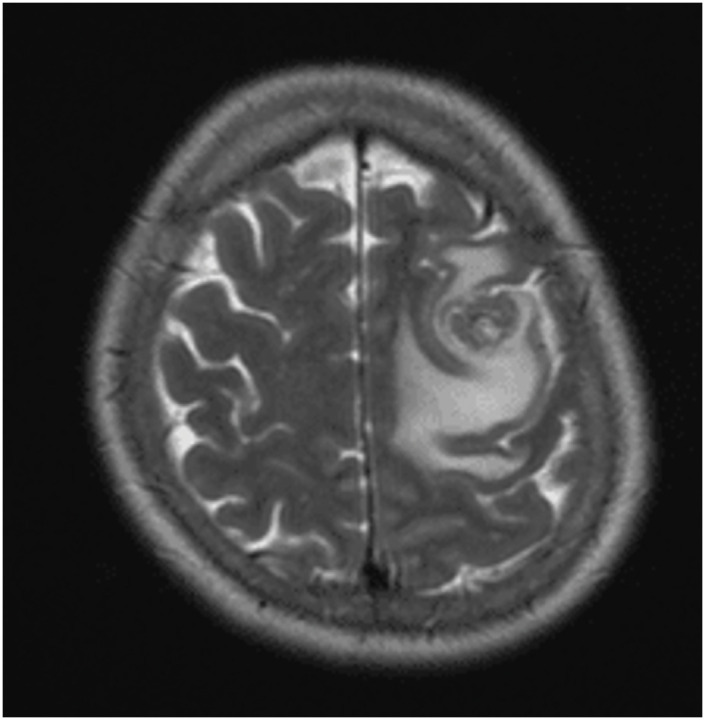
T2-weighed MRI demonstrated an 18-mm nodule in the left cerebral lobe with surrounding edema.

## DISCUSSION

This case involved a patient who developed a solitary subcutaneous gluteal metastasis following curative resection of stage IIIC cecal cancer and who subsequently presented with brain metastasis after resection of the gluteal lesion. Cutaneous and subcutaneous metastases from visceral malignancies typically arise near the primary tumor site. However, their occurrence at distant sites, representing true systemic dissemination, is exceedingly rare. Although the metastatic pathway remains unclear, the clinical course of this case provides valuable insights into the possible mechanisms underlying distant subcutaneous spread.

The most common primary tumors associated with cutaneous metastasis are lung cancer in men and breast cancer in women, whereas visceral tumors account for only 0.7%–9%.^5)^ For colorectal cancer, the liver (75%), lungs (15%), bones (5%), and central nervous system (5%) are the predominant metastatic sites.^6)^ The reported frequencies of cutaneous and subcutaneous colorectal cancer metastases are 2.3%–4.4%,^1,7)^ making them relatively uncommon. Among cases of cutaneous or subcutaneous metastasis, lesions occurring near the surgical site are generally regarded as tumor implantations in the skin damaged by surgical procedures.^1–3)^ It is not unusual for locally recurrent rectal cancer to form lesions in the gluteal or perineal regions, as rectal cancer may require perineal surgical approaches and possesses lymphatic drainage pathways that pass through the perineum toward the inguinal region, potentially facilitating tumor spread or recurrence along this route. In contrast, metastases arising at distant sites from the primary tumor are considered true distant metastases and are extremely rare. Subcutaneous colon cancer metastases to the gluteal or perineal region are classified as distant metastases, owing to their anatomical distance from the primary lesion.^4)^ To the best of our knowledge, including the present case, only 6 cases of solitary cutaneous or subcutaneous colon cancer metastasis to the gluteal or perineal region have been reported worldwide, from colon cancers located from the cecum to the rectosigmoid (**Table 1**).^4,6,8–10)^ Among these, the primary sites were the right-sided colon (cecum to transverse colon) in 3 cases and the left-sided colon (descending colon to rectosigmoid) in 3 cases. Notably, the interval from initial surgery to the development of subcutaneous metastasis was short in all cases, ranging from 3 to 10 months. Extensive lymph node metastasis was observed in 4 cases, and lymphatic invasion was present in 5 cases. All gluteal and perineal metastases were surgically resected; 4 patients remained recurrence-free, whereas 2, including the present case, subsequently developed distant recurrences.

**Table 1:** Summary of reported cases of solitary cutaneous or subcutaneous metastasis at the gluteal or perineal region after curative resection of colon cancer

Case numbers	Author (country)	Years	Age/sex	Metastatic region	Duration from primary tumor resection to metastasis (months)	Primary tumor site/differentiation	pT/pN	Lymphatic invasion/venous invasion	Adjuvant chemotherapy after primary tumor resection	Regimen	Resection of the metastatic region	Recurrence/Prognosis after resection of metastatic region (follow-up months)
1	Okazaki et al.^8)^ (Japan)	2003	76/female	Right perineal cutaneous	3	Sigmoid colon/ well differentiated	T2/ N0	Mild/ absent	No	—	Yes	No recurrence/ alive (24 months)
2	Matsuoka et al.^9)^ (Japan)	2004	84/female	Left perineal subcutaneous	4	Ascending colon/ mucinous	T3/ N2	Moderate/ absent	No	—	Yes	No recurrence/ alive (12 months)
3	Gomes et al.^6)^ (Brazil)	2012	78/female	Left gluteal cutaneous	8	Ascending colon/ poorly differentiated	T3/ N1	Present/ present	Yes	Capecitabine (2 cycles)	Yes	No recurrence/ alive (5 months)
4	Lee and Lee^4)^ (Korea)	2013	49/male	Posterior perineal subcutaneous	9	Sigmoid colon/ moderately differentiated	T2/ N2	Absent/ absent	Yes	Capecitabine (8 cycles)	Yes	Lung (8 months)/ death (30 months)
5	Hosaka^10)^ (Japan)	2023	56/female	Right perineal subcutaneous	9	Rectosigmoid/ moderately differentiated	T3/ N0	Mild/ mild	Yes	Capecitabine (1 cycle) → tegafur uracil and levofolinate calcium (5 cycles)	Yes	No recurrence/ alive (27 months)
6	Present case (Japan)	2026	58/female	Left gluteal subcutaneous	10	Cecum/well differentiated	T4a/ N2	Moderate/ mild	Yes	CAPOX (4 cycles)	Yes	Brain (9 months)/ alive (14 months)

CAPOX, capecitabine and oxaliplatin

Possible routes of distant metastasis to the skin include hematogenous, lymphatic, and peritoneal dissemination. However, cutaneous metastasis from visceral malignancies is predominantly hematogenous in origin.^1)^ In a previous report of 413 patients with metastatic colorectal cancer, 18 (4.4%) developed cutaneous metastases, most of which occurred at abdominal incision sites^1)^ and were presumed to result from surgical implantation rather than true distant metastasis. In contrast, subcutaneous metastases in the gluteal or perineal region from colorectal cancer, given their anatomical distance from the primary tumor, are regarded as true distant metastases. Such cases are frequently associated with extensive lymph node involvement and lymphatic invasion, suggesting the possibility of lymphatic dissemination.^11,12)^ Anatomically, colorectal cancer typically spreads along mesenteric and pelvic lymphatic pathways; however, in the presence of extensive nodal involvement or lymphatic obstruction, aberrant or retrograde lymphatic flow may allow tumor cells to reach atypical regions such as the gluteal area.^13)^ Therefore, lymphatic spread to the gluteal region represents a plausible mechanism.

However, in the present case, brain metastasis developed 9 months after resection of a solitary gluteal subcutaneous metastasis. Similarly, Lee et al. reported pulmonary metastasis following resection of a solitary perineal subcutaneous metastasis.^4)^ In both cases, recurrence occurred in organs typically associated with systemic hematogenous dissemination, such as the brain and lungs. These observations suggest that subcutaneous metastases in the gluteal or perineal region may, in some cases, reflect early systemic dissemination. Thus, while lymphatic spread cannot be excluded, hematogenous dissemination should also be considered as a plausible and potentially predominant pathway.

From a biological perspective, the sequential development of gluteal and brain metastases is noteworthy. Brain metastasis from colorectal cancer is relatively uncommon, partly due to the protective role of the blood–brain barrier. One possible explanation is that the gluteal lesion acted as a “stepping stone” for further dissemination, as the subcutaneous microenvironment may support tumor cell survival, adaptation, and intravasation. Alternatively, this pattern may reflect the presence of tumor cell subclones with high metastatic potential capable of overcoming biological barriers, including the blood–brain barrier. Although the precise mechanism remains unclear, these hypotheses underscore the biological heterogeneity of colorectal cancer and the potential for aggressive behavior in cases with atypical metastatic patterns.

Several reports have demonstrated that resection of solitary metachronous gluteal or perineal metastases can yield favorable outcomes. However, given that such lesions may represent an early phase of systemic dissemination, careful postoperative surveillance is warranted even after curative resection. Clinicians should recognize that gluteal or perineal subcutaneous metastasis may not always represent localized disease but may instead reflect underlying systemic spread. In this context, emerging surveillance tools such as circulating tumor DNA may be useful for detecting occult systemic disease.

## CONCLUSIONS

Solitary gluteal or perineal subcutaneous metastases from colon cancer are rare and may represent an early manifestation of systemic disease. Despite the unclear metastatic pathway, these lesions may indicate aggressive tumor biology with a risk of subsequent distant spread, warranting careful postoperative surveillance.

## DECLARATIONS

### Availability of data and materials

The data supporting the findings of this study are available from the corresponding author upon reasonable request.
